# Accumulation of ether phospholipids in induced pluripotent stem cells and oligodendrocyte‐lineage cells established from patients with Sjögren‐Larsson syndrome

**DOI:** 10.1111/cga.12587

**Published:** 2024-12-01

**Authors:** Yu Yamaguchi, Hironobu Okuno, Suzumi Tokuoka, Yoshihiro Kita, Tsukasa Sanosaka, Jun Kohyama, Kenji Kurosawa, Norio Sakai, Fuyuki Miya, Takao Takahashi, Kenjiro Kosaki, Hideyuki Okano

**Affiliations:** ^1^ Department of Genetics Gunma Children's Medical Center Shibukawa Japan; ^2^ Department of Physiology Keio University School of Medicine Tokyo Japan; ^3^ Department of Pediatrics Keio University School of Medicine Tokyo Japan; ^4^ Center for Medical Genetics Keio University School of Medicine Tokyo Japan; ^5^ Department of Pediatrics and Adolescent Medicine Tokyo Medical University Tokyo Japan; ^6^ Department of Lipidomics, Graduate School of Medicine The University of Tokyo Tokyo Japan; ^7^ Life Sciences Core Facility, Graduate School of Medicine The University of Tokyo Tokyo Japan; ^8^ Division of Medical Genetics Kanagawa Children's Medical Center Yokohama Japan; ^9^ Division of Health Sciences Osaka University Graduate School of Medicine Osaka Japan; ^10^ Center for Developmental Neurology Shin‐yurigaoka General Hospital Kawasaki Japan; ^11^ Keio University Regenerative Medicine Research Center Kawasaki Japan

**Keywords:** ether phospholipid, FALDH, induced pluripotent stem cells, oligodendrocytes, Sjögren‐Larsson syndrome

## Abstract

Sjögren‐Larsson syndrome (SLS) is an autosomal recessive leukodystrophy characterized by ichthyosis, intellectual disability, and progressive spastic paralysis caused by biallelic pathogenic variants in the *ALDH3A2* gene that encodes the fatty aldehyde dehydrogenase, fatty aldehyde dehydrogenase (FALDH); FALDH catalyzes several metabolic reactions involved in fatty aldehyde oxidation. Only a few studies have been performed to determine the lipid profile of patients with SLS. In a previous postmortem study of the brain of a 65‐year‐old patient with SLS, lipidomic analysis revealed an accumulation of long‐chain unsaturated ether lipid species in the white matter and gray matter. In the present study, we established a disease model using patient‐derived neuronal and oligodendrocyte lineage cells to analyze the lipid metabolism and gene expression profiles in SLS. To achieve this, we generated induced pluripotent stem cells (iPSCs) from two patients with the SLS phenotype carrying previously known *ALDH3A2* pathogenic variants: One was a compound heterozygote (c.1339A>G:p.(Lys447Glu) and c.57_132dup:p.(Ile45Serfs*34)) and the other was a homozygote (c.1339A>G: p.(Lys447Glu)). The FALDH activity was almost zero in the SLS‐iPSC lines established from both patients. Phospholipid analysis of neurospheres, and oligospheres (spheres enriched with oligodendrocyte‐lineage cells) derived from the iPSCs by liquid chromatography‐mass spectrometry showed accumulation of ether phospholipids in the Sjögren‐Larsson patient‐derived neurospheres and oligospheres. The results are consistent with the previously reported accumulation of ether lipids in the postmortem brain tissue of an SLS patient. Therefore, iPSCs and iPSC‐derived neurospheres and oligospheres established from SLS patients can be useful tools for future pathological analysis of the central nervous system pathophysiology in SLS.

## 1 INTRODUCTION

Sjögren‐Larsson syndrome (SLS) is a rare autosomal recessive leukodystrophy characterized by congenital ichthyosis and neurological manifestations such as spasticity and intellectual disabilities.^1^ SLS is caused by biallelic pathogenic loss‐of‐function variants of the *ALDH3A2* gene^2^ that encodes the NAD^+^‐dependent enzyme, fatty aldehyde dehydrogenase (FALDH). FALDH is expressed in the endoplasmic reticulum (ER) and catalyzes the oxidation of various aliphatic fatty aldehydes to fatty acids, including sphingosine‐1‐phosphate, dietary phytanic acid, leukotriene B_4_, and very long chain fatty acids.^3^

Among the various leukodystrophies, SLS is characterized by a skin manifestation, namely, ichthyosis. An abnormal lamellar structure has been reported in the skin of patients with SLS.^4^ Knowledge of the pathophysiology in the central nervous system of patients with SLS is currently limited. Magnetic resonance imaging (MRI) of the brains of SLS patients shows hypomyelination in the cerebral white matter, and magnetic resonance spectroscopy (MRS) shows abnormal peaks indicative of lipid accumulation.^5^ In a postmortem study of the brain of a 65‐year‐old patient with SLS, lipidomic analysis revealed an accumulation of ether phospholipid species in the white matter, but no increase of plasmalogen, a class of ether phospholipids reported to be abundant and bioactive in human brain tissues.^6^ This observation provided critical insight into the pathogenesis of SLS, but it would be unreasonable to generalize the findings of lipidomic profiling of a single patient's postmortem brain. Therefore, in vitro modeling of the central nervous system in SLS was needed for further investigation and therapeutic research. In the present study, we established an in vitro cellular disease model by generating induced pluripotent stem cells (iPSCs) from two SLS patients. The FALDH activity in both the SLS‐iPSC lines was almost zero and significantly decreased as compared with that in the control iPSC lines. We further differentiated these cells into oligodendrocyte‐like cells,^7^ and performed lipidomic analysis of the phospholipid profile in these cells.

## 2 MATERIALS AND METHODS

### 2.1 Clinical characteristics of SLS patients

All the experimental procedures were approved by the Ethics Committee of Keio University School of Medicine (approval number: 20080016) and the Research Ethics Committee of The University of Tokyo (approval number: 11793), and appropriate informed consent was obtained from the parents of the patients. We enrolled two SLS patients, Patient 1 (1SLS) and Patient 2 (2SLS), with biallelic pathogenic variants of the *ALDH3A2* gene who exhibited the typical phenotype of SLS.

**FIGURE 1:**
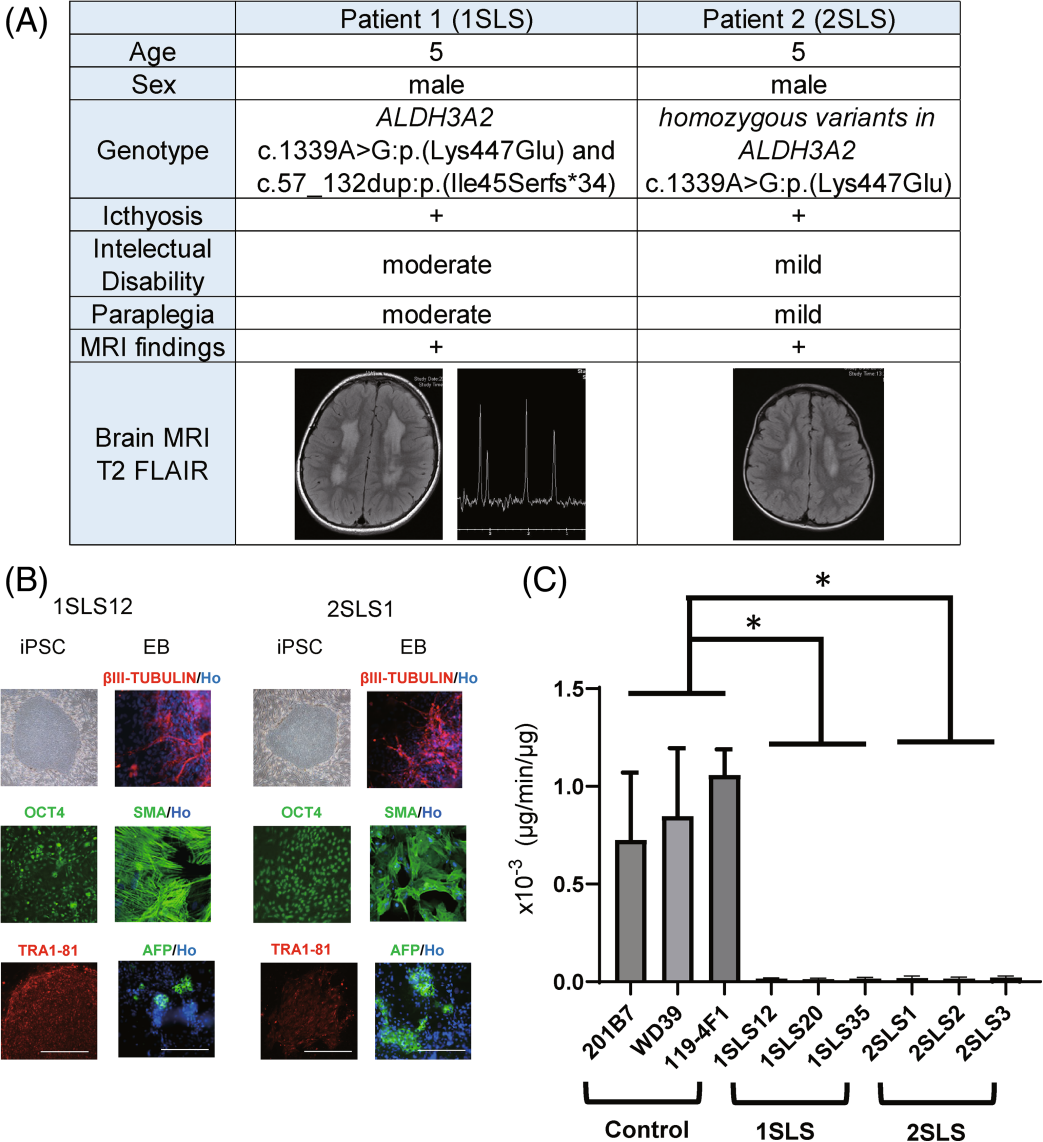
Characterization of Sjögren‐Larsson (SLS) syndrome patients' specific iPSCs. (A) Summary of two patients with Sjögren‐Larsson syndrome. Both patients showed typical cutaneous and neurological symptoms. iPSC lines (1SLS12, 1SLS20, 1SLS35, and 1SLS36) were generated from a 5‐year‐old Japanese boy diagnosed as having Sjögren‐Larsson syndrome (SLS). The patient was a compound heterozygote for missense and frameshift mutations in the *ALDH3A2* gene. Another set of 4 iPSC lines (2SLS1, 2SLS2, 2SLS3, 2SLS4) were generated from another 5‐year‐old Japanese boy with SLS carrying homozygous missense mutations in the *ALDH3A2* gene. (B) Microscopic images of the iPSCs. The overall shape of the iPSCs was comparable to that of human ESCs. All of the cell lines expressed the human pluripotent stem cell markers: OCT4, Tra1‐81. Scale bar, 200 μm. Embryoid bodies (EBs) differentiated from iPSCs expressed three germ layer markers: AFP (an endoderm marker), SMA (a mesoderm marker), and βIII‐TUBULIN (an ectoderm marker). Ho; Hoechst. (C) FALDH activity assay of iPSCs derived from the control and SLS patients. Deuterium‐labeled hexadecanol was reacted with the protein supernatants extracted from the control and SLS‐derived iPSCs in the presence of nicotinamide adenine dinucleotide (NAD^+^), and the amount of fatty acids produced per volume of protein supernatant (μg) was measured by LCMS; the FALDH enzyme activity (μg/min/μg) was thus quantified. A significant decrease in enzyme activity was observed in the iPSCs derived from both the SLS patients (1SLS, 2SLS). Statistically significant differences are indicated. (*adjusted *p* < 0.05; Dunnett's multiple comparisons test, technical replicates; 3) Error bars represent ± standard deviation (SD).

Patient 1 was a 5‐year‐old Japanese boy who was born at term with ichthyosis and developed paraplegia at the age 2 years (Figure 1A, left). He was unable to stand on his own and was only able to say a few words even by 5 years of age. Brain MRI at the age of 3 years showed diffuse and symmetrical high signal intensity bilaterally in the periventricular white matter on T2‐weighted images, and MR spectroscopy showed an abnormal peak at 1.3 ppm, which is specific for SLS.^8^ He had compound heterozygous variants of *ALDH3A2* (NM_000382.3:c.1339A>G:p.(Lys447Glu), c.57_132dup:p.(Ile45 Serfs*34)), which have previously been as being pathogenic.^9^

Patient 2 was also a 5‐year‐old Japanese boy (Figure 1A, right) who developed scaly skin lesions and was also diagnosed with developmental delay in late infancy. At the age of 5 years, he could walk with mild spasticity and had mild intellectual disability. His brain MRI showed bilateral high signal intensity on T2‐weighted images. He had a homozygous non‐synonymous variant of *ALDH3A2* (c.1339A>G:p.(Lys447Glu)).

### 2.2 Generation of iPSCs from the fibroblasts and peripheral blood mononuclear cells (PBMCs)

iPSCs were generated from skin fibroblasts derived from patient 1 (1SLS) and two healthy volunteers by retroviral transduction of four transcription factors (KLF4, OCT4, SOX2, and c‐MYC)^10^ and from PBMCs derived from patient 2 (2SLS) by transduction of episomal plasmids encoding transcription factors (OCT3/4, SOX2, KLF4, L‐MYC, LIN28) and p53‐shRNA by electroporation.^11^, ^12^ Seven days after the transduction of reprogramming factors, we replated the cells onto mitomycin C‐treated SNL feeder cells, and iPSCs emerged by 2–3 weeks after replating (Figure 1B).

### 2.3 Measurement of the FALDH activity in the iPSCs

The FALDH enzyme activity was measured in the patient‐derived iPSCs (Figure 1C). The iPSCs were cultured in six‐well plates without feeder cells and collected after washing in phosphate buffer solution (PBS). They were then centrifuged, and the pellets were resuspended in 150 μL of ice‐cold buffer containing 100 mM Tris–HCl (pH 7.4) and 250 mM sucrose and cOmplete™, an ethylenediaminetetraacetic acid‐free proteinase inhibitor cocktail (Roche, Mannheim, Germany) and then sonicated on ice. After centrifugation at 800×*g* for 5 min, the post‐nuclear supernatant (containing the cytosol and microsomal fraction) was collected. The reaction mixture contained 50 mM Tris–HCl (pH 8.2), 1 mM NAD^+^ (Sigma Aldrich, MO, USA), 40 μM 16:0 aldehyde‐d9 (Avanti Polar Lipids, AL, USA), 4 μg/mL 15:0 fatty acid, and 5 μg cellular proteins in a total volume of 50 μL. Enzyme reactions were performed on a thermal block at 37°C. After 10 min of incubation, 300 μL of methanol was added to stop the reaction. 15:0 fatty acid and 16:0‐d9 fatty acid were measured by LCMS‐8080 (Shimadzu, Kyoto, Japan). The amounts of 16:0‐d9 fatty acid as an enzyme product were measured by applying the standard curve for 15:0 fatty acid. Enzyme assay without NAD^+^ was also performed for all the samples and there were no detectable enzyme products.

### 2.4 Generation of neurospheres from the iPSCs

**FIGURE 2:**
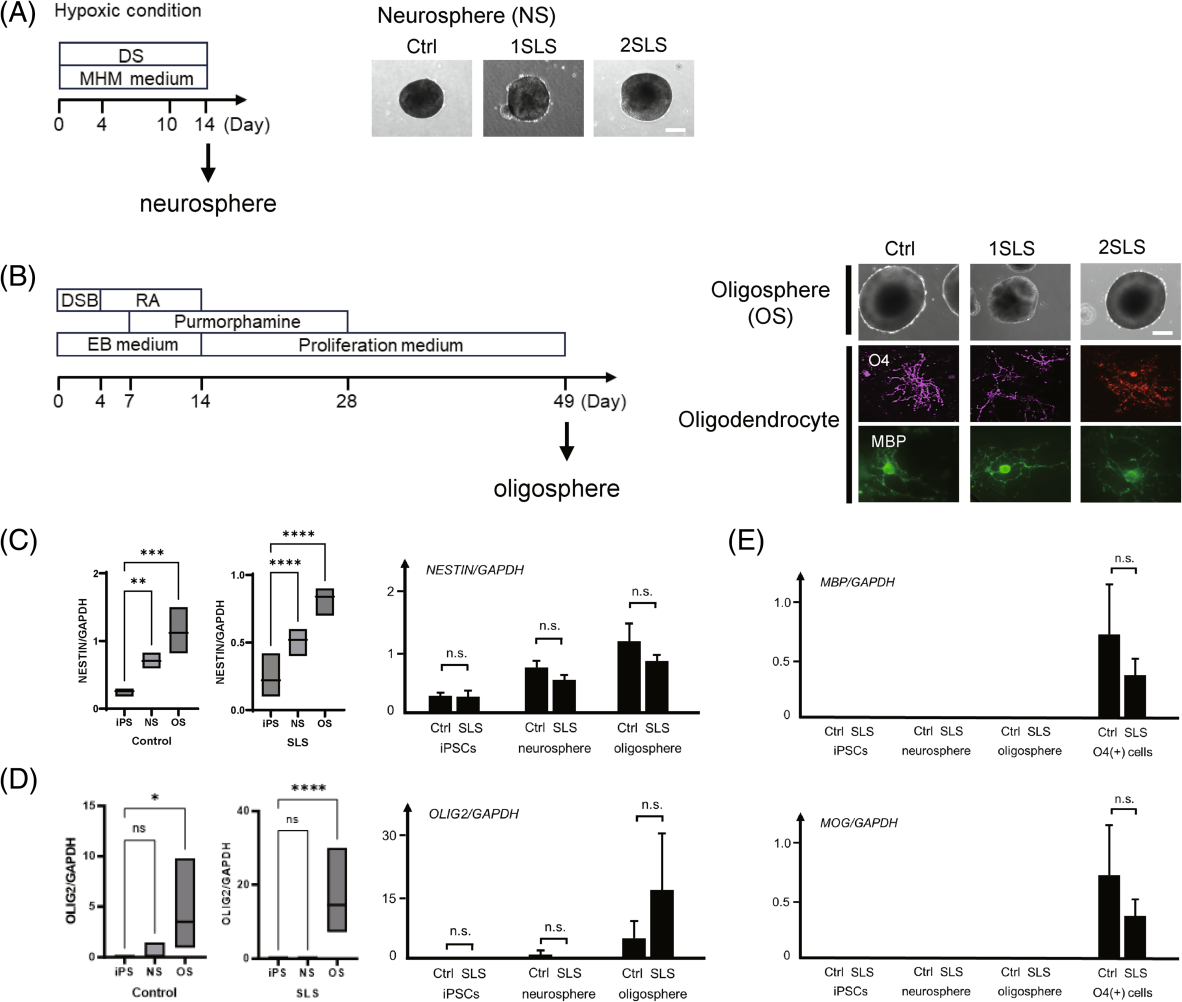
Induction of oligospheres from iPSCs. (A) Schematic protocol of generation of neurospheres from iPSCs and morphology of the neurospheres. DS; dual SMAD inhibition, CHIR99021 and SB431542. Bar: 200 μm. (B) Schematic protocol of oligodendrocyte differentiation of iPSCs. On day 49, oligospheres were seeded and cultured for adhesion. After approximately 40 days, differentiation into O4‐positive and MBP‐positive oligodendrocytes was confirmed. DSB: Dorsomorphin, SB431542, and BIO (a GSK3 inhibitor); RA: Retinoic acid; EB, embryoid body. Bar: 200 μm. (C) (Left) Comparison of the *NESTIN* mRNA expression levels normalized to *GAPDH* expression in the iPSCs, neurospheres, and oligospheres by quantitative RT‐PCR. **p < 0.01, ***p < 0.001, ****p < 0.0001; Dunnett's multiple comparisons test. (Right) Comparison of the *NESTIN* mRNA expression levels normalized to the expression of *GAPDH* between the control and SLS iPSCs, neurospheres and oligospheres. n.s.: No significant; *t*‐test. (D) (Left) Comparison of the *OLIG2* mRNA expression levels normalized to the *GAPDH* expression in the iPSCs, neurospheres, and oligospheres by quantitative RT‐PCR. ns; no significant, *p < 0.05, ****p < 0.0001; Dunnett's multiple comparisons test. (Right) Comparison of the *OLIG2* mRNA expression levels normalized to the *GAPDH* expression between the control and SLS iPSCs, neurospheres, and oligospheres. ns.: Not significant; *t*‐test. (E) Comparison of the *MAP* and *MOG* mRNA expression levels normalized to the *GAPDH* expression in the iPSCs, neurospheres, oligospheres and O4 positive cells by quantitative RT‐PCR. (ns.: Not significant; *t*‐test.) In C, D and E., control: 201B7, WD39,119‐4F1 and 119‐4F2; SLS: 1SLS12, 1SLS20, 1SLS35, 1SLS36, 2SLS1, 2SLS2, 2SLS3, and 2SLS4.

We generated neurospheres from the iPSC lines in accordance with a previously reported protocol (Figure 2A).^13^, ^14^ Briefly, we dissociated iPSCs, cultured in human iPS medium containing SB431542 (a transforming growth factor β [TGF‐β] receptor inhibitor) and CHIR99021 (a GSK3β inhibitor) for more than 5 days, into single cells, then cultured them in MHM medium with B27, bFGF, hLIF, CHIR99021, and SB431542 under hypoxic conditions. After 14 days in culture, neurospheres capable of neuronal differentiation were formed and collected.

### 2.5 Generation of oligospheres from iPSCs and differentiation into oligodendrocytes in vitro

We generated oligodendrocyte progenitor cell (OPC)‐enriched spheres, that is, oligospheres, from the iPSCs lines in accordance with a previously reported protocol (Figure 2B).^7^ Briefly, dorsomorphin (a bone morphogenetic protein signaling inhibitor), SB431542, and BIO (a GSK3 inhibitor) were added during embryoid body (EB) formation. Next, we added retinoic acid for caudalization, and purmorphamine (Sonic hedgehog‐signal agonist) for ventralization during EB formation until its dissociation. We then cultured the dissociated EBs in suspension to form neurospheres in a proliferation medium supplemented with factors that promote the commitment and proliferation of oligodendrocyte lineage cells. For adherent differentiation, oligospheres were cultured in a differentiation medium supplemented with factors that promote the commitment of oligodendrocyte lineage cells (Figure 2B). The adherent culture was a mixture of oligodendrocyte lineage cells as well as other glial progenitors and neurons. After 4 weeks of oligosphere adherent culture, O4^+^ cells were isolated using the magnetic cell sorting (MACS) method with an anti‐O4 antibody (Miltenyi Biotec, Bergisch Gladbach, Germany), followed by analysis for the expression of mature oligodendrocyte markers.

### 2.6 RT‐PCR and primer information

RNAs were extracted from the cultured cells and real‐time quantitative reverse transcription polymerase chain reaction (RT‐PCR) was performed as previously described, using SYBR Premix ExTaq II and the MX3000P Real‐Time PCR system (Figure 2C).^7^ The amount of cDNA was normalized to that of human‐specific beta‐actin messenger RNA (mRNA). The applied primer sequences are listed below:

MBP Sense: 5′‐GGGCATCCTTGACTCCATC‐3′
MBP Antisense: 5′‐TCCTTGTACATGTTGCACAGC‐3′
MOG Sense: 5′‐TCACTGTTGGCCTCATCTTC‐3′
MOG Antisense: 5′‐CAAAAGTCCGGTGGAGATTC‐3′
NESTIN Sense: 5′‐TGCGGGCTACTGAAAAGTTC‐3′
NESTIN Antisense: 5′‐TGGGAGCAAAGATCCAAGAC‐3′
OLIG2 Sense: 5′‐GTTCTCCCCTGAGGCTTTTC‐3′
OLIG2 Antisense: 5′‐AGAAAAAGGTCATCGGGCTC‐3′
GAPDH Sense: 5′‐ACCCAGAAGACTGTGGATGG‐3′
GAPDH Antisense: 5′‐TCTAGACGGCAGGTCAGGTC‐3′

### 2.7 Analysis of the phospholipid profiles

The samples to be analyzed were washed twice with PBS and the phospholipids contained therein were extracted with methanol. Liquid chromatography‐mass spectrometry (LC–MS) systems were used for profiling analysis of phosphatidylcholines (PC) and phosphatidylethanolamines (PE). Nexera UHPLC (Shimadzu) was connected to an LCMS‐8040 (Shimadzu) or LCMS‐8050 (Shimadzu) mass spectrometer. An Acquity BEH C8 column (2.1 × 100 mm) was used at 45°C for reversed‐phase chromatographic separation of the phospholipids. Neurosphere samples were analyzed using a binary gradient system (mobile phase A: 20 mM NH_4_HCO_3_ in water; mobile phase B: acetonitrile; 0.4 mL/min flow rate) using the following time program (time (%A/%B)): 0 min (80/20)–20 min (5/95)–50 min (5/95)–50.1 min (80/20)–55 min (80/20). Oligospheres and iPS cells were analyzed using a ternary gradient separation system (mobile phase A: 5 mM NH_4_HCO_3_ in water; mobile phase B: acetonitrile; mobile phase C: 2‐propanol; 0.35 mL/min flow rate) using the following time program (time (%A/%B/%C)): 0 min (75/20/5)–20 min (20/75/5)–40 min (20/5/75)–45 min (5/5/90)–50 min (5/5/90)–50.1 min (75/20/5)–55 min (75/20/5).

For selective detection of PC and PE phospholipids, MRM transitions of [M + H]^+→^184 and [M + H]^+→^[M + H‐141]^+^ were used (Table S1). Peak areas of individual PC and PE molecular species were normalized to the total PC area and total PE area, respectively. Peak areas were integrated using the TRACES software^14^ and statistical calculation was performed using the R software (version 4.3).

The results of comparison of the phospholipid profiles were expressed as volcano plots, where the log2 ratio (control versus SLS) and ‐log10 *t*‐test *p*‐value of a particular lipid were plotted on the horizontal and vertical axis, respectively. The R package ggplot2 was used for the construction of the volcano plots.

A heatmap was generated for phospholipids with raw *t*‐test *p*‐values (less than 0.05) in the volcano plot. The cells of the heatmap were colored according to the row *z*‐score value of the relative abundance obtained among the samples.

### 2.8 RNA‐seq

Total RNA was extracted using an RNeasy Mini Kit (QIAGEN, Hilden, Germany). Poly(A)^+^ RNA was selected and converted to a library of cDNA fragments (200–250 bp) with adapters attached at both ends for sequencing using a TruSeq Stranded mRNA LT Sample Prep Kit Set (Illumina, CA, USA) in accordance with the manufacturer's instructions. The library was quantified using the KAPA library quantification kit (KAPA Biosystems, MA, USA) and sequenced on the HiSeq 2,500 (Illumina) in rapid mode. Base calling and chastity filtering were performed using the Real‐Time Analysis Software version 1.18.61. The resulting reads were mapped to the UCSC human genome 19 using Sailfish version 0.7.6 under default settings. Differential expression analysis was performed using the R software package “TCC.”

## 3 RESULTS

### 3.1 Generation and characterization of control and Sjögren Larsson syndrome iPSCs

We established 4 iPSC lines for each SLS patient. The iPSC lines from SLS patient 1 (1SLS12, 1SLS20, 1SLS35, and 1SLS36) were established from the skin fibroblasts, and those from SLS patient 2 (2SLS1, 2SLS2, 2SLS3, and 2SLS4) were established from the PBMCs, as previously described.^11^, ^12^ Control iPSC lines were previously generated from the skin fibroblasts of a 34‐year‐old healthy woman (201B7), 16‐year‐old healthy woman (WD39)^10^, ^15^, ^16^ and a 6‐year‐old healthy Japanese boy (119‐4F1 and 119‐4F2).^17^ Each iPSC line showed a typical human embryonic stem cell (ESC) morphology with a high nuclear‐cytoplasmic ratio and formed discoid colonies (Figure 1B), expression of pluripotent stem cell markers as determined by immunocytochemistry (OCT4 and TRA1‐81. Figure 1B), silencing of transgenes as shown by quantitative RT‐PCR (data not shown), and the ability to differentiate into endodermal lineage (AFP), mesodermal lineage (SMA) and ectodermal lineage (βIII‐TUBULIN) as shown by the embryoid formation assay^7^, ^18^ (Figure 1B). We also confirmed a normal karyotype of these iPSC lines (Figure S1).

Since FALDH enzyme deficiency is critical for the pathology of SLS^9^, we analyzed whether the activity of FALDH, that is, fatty aldehyde dehydrogenase, is decreased in SLS‐iPSCs. The FALDH enzyme activity in the control and SLS‐iPSCs was quantified by LC–MS to measure the amount of deuterium‐labeled fatty acid, which is a product of the oxidation reaction of deuterium‐labeled fatty aldehyde. The results revealed significantly decreased FALDH activity in each of the SLS‐iPSC lines examined as compared with that in the control iPSC lines (*t*‐test, *p* < 0.0001). In fact, the FALDH activity was almost zero in the SLS‐iPSC lines (Figure 1C).

### 3.2 Induction of oligospheres from both control‐ and SLS‐ iPSCs

We differentiated iPSCs into oligodendrocytes via oligospheres as previously reported^7^, ^18^ (Figure 2B). Gene expression analysis was performed to understand the properties of differentiated oligodendrocytes. The oligospheres collected on day 49 were differentiated into oligodendrocytes by 2 weeks of adhesion culture. Immunocyte staining showed that the obtained cells expressed the oligodendrocyte‐lineage markers O4 and MBP (Figure 2B). Gene expression analysis was performed by quantitative PCR to determine the characteristics of the oligospheres used in the subsequent analyses.

First, as shown in Figure 2C, we analyzed *NESTIN*, which is known to be expressed in neural stem cells and neural precursor cells,^19^ and found that *NESTIN* gene expression was significantly higher in the neurospheres than in iPSCs, and even higher in oligospheres. However, there was no difference in the gene expression between Control and SLS iPSCs, neurospheres, and oligospheres (Figure 2C).

Next, as shown in Figure 2D, we analyzed the gene expression of *OLIG2*, which is known to be expressed in oligodendrocyte lineage cells. The expression level of *OLIG2* was significantly higher in the oligospheres than in the iPSCs and neurospheres both in control lines and in SLS patient lines, while there was no difference in the gene expression between the Control and SLS iPSCs, neurospheres, and oligospheres.

In addition, as shown in Figure 2E, gene expression levels of *MBP* and *MOG*, which are oligodendrocyte markers (myelin‐related structural proteins), were analyzed in O4‐positive cells obtained after 2 more weeks of adhesion culture of the analyzed oligospheres. No *MBP* or *MOG* gene expression could be detected in any of the iPSCs, neurospheres or oligospheres, while the genes were expressed in both control and SLS oligodendrocytes; however, no difference in the gene expression level was found between the control and SLS oligodendrocytes.

Finally, we confirmed that the oligospheres differentiated from iPSCs were actually OPC‐enriched spheres.

### 3.3 Phospholipid analysis revealed the accumulation of ether phospholipids in SLS‐patients‐derived iPSCs neurospheres, and oligospheres

We performed targeted LC–MS analysis of PC and PE, the major lipid subclasses containing ether phospholipids (especially plasmalogens) in the control and SLS patients‐derived iPSCs, iPSC‐derived neurospheres, and iPSC‐derived oligospheres.

**FIGURE 3:**
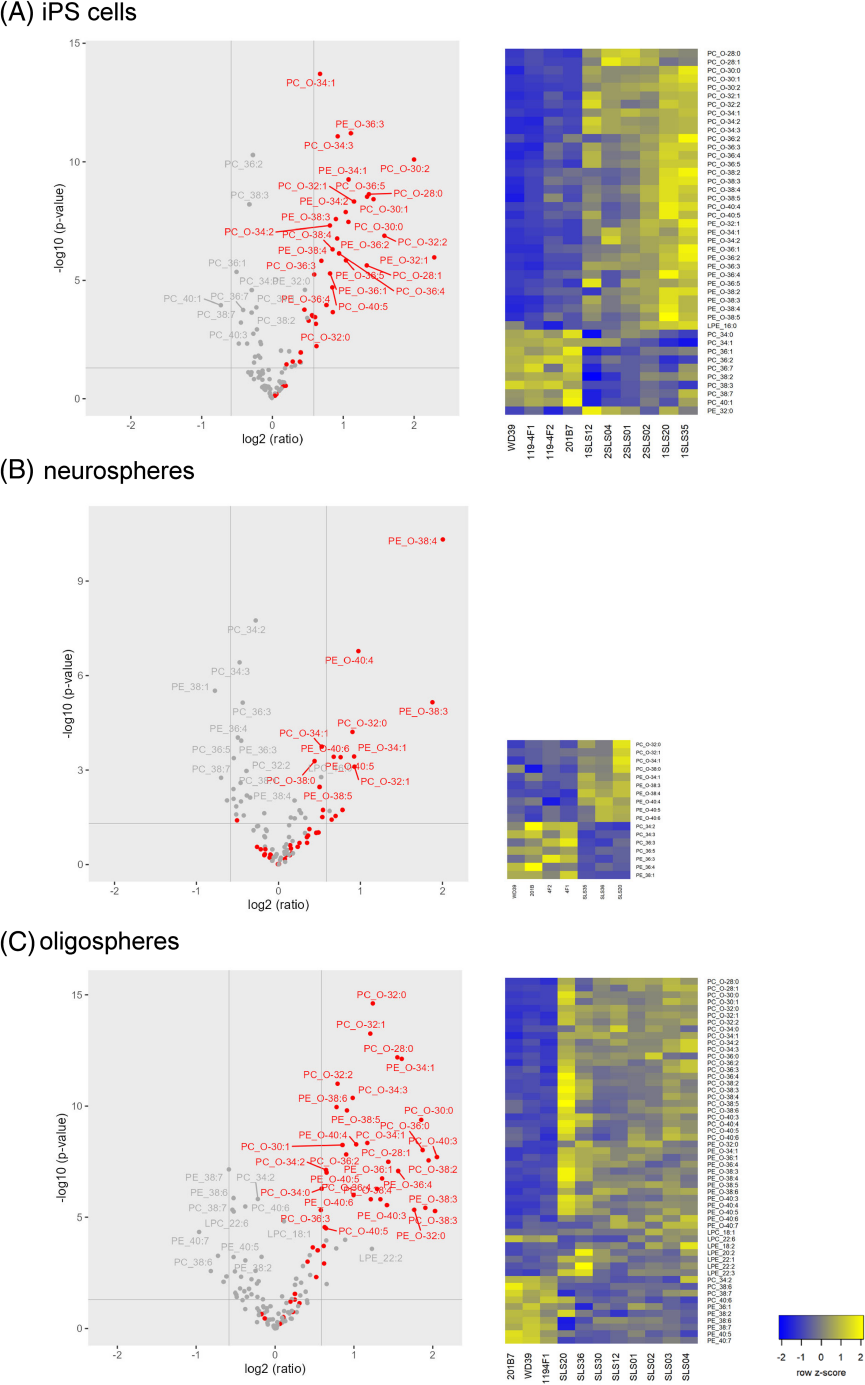
Predominance of ether phospholipids over diacyl phospholipids in the SLS‐derived cells. The predominance of ether phospholipids over diacyl phospholipids was evident in the iPSCs and oligodendrocytes, and a similar finding was noted in the neurospheres. Comparisons between the control (WD39, 201B7, 119‐4F1, 119‐4F2) and SLS cells (1SLS12, 1SLS20, 1SLS35, 2SLS01, 2SLS02, 2SLS04) are presented as volcano plots, where the horizontal axis indicates the log intensity ratio of the percent relative abundance between SLS‐derived cells and control‐derived cells (i.e., control versus SLS), with the vertical axis indicating statistical significance (*p*‐values with a threshold of 0.05). Each circle represents one lipid species. Red circles indicate ether phospholipids and gray circles indicate diacyl phospholipids. Heatmap of the *Z*‐scores of the percent relative abundance of each phospholipid. The four columns on the left show the findings for cells obtained from 4 normal individuals and the six columns on the right show the results for the cells obtained from the 2 patients with SLS, SLS patient 1 and SLS patient 2. The listed lipid species in the heatmaps were selected based on their low ‐log_10_ (*p*‐value) (more than 1) when constructing the volcano plot. The cells of the heatmap are colored according to the row *z*‐score value of the percent relative abundance obtained among 10 samples (4 from normal and 6 from SLS patients). Left, 4 columns (normal control), and right, 6 columns (SLS samples), are ordered within each group according to the clustering method using the row *z*‐scores. The volcano plots and heatmap were prepared for iPSCs (A), neurospheres (B), and oligospheres (C).

Comparison of the relative abundance of phospholipid species showed that ether phospholipids tended to be relatively abundant in cells generated from SLS patients as compared with those generated from healthy controls (Figure 3A–C). The predominance of ether phospholipids over diacyl phospholipids was evident in iPSCs and oligospheres, and a similar trend was observed in the neurospheres. The Heatmap also supported the notion that cells from SLS patients tended to show an abundance of ether phospholipids (Figure 3A–C).

### 3.4 Aberrant gene expression related to ether phospholipid metabolism in the SLS‐oligospheres

RNAseq was performed to elucidate the pathology caused by FALDH enzyme deficiency at the gene expression level by comprehensively analyzing the gene expression profile.

**FIGURE 4:**
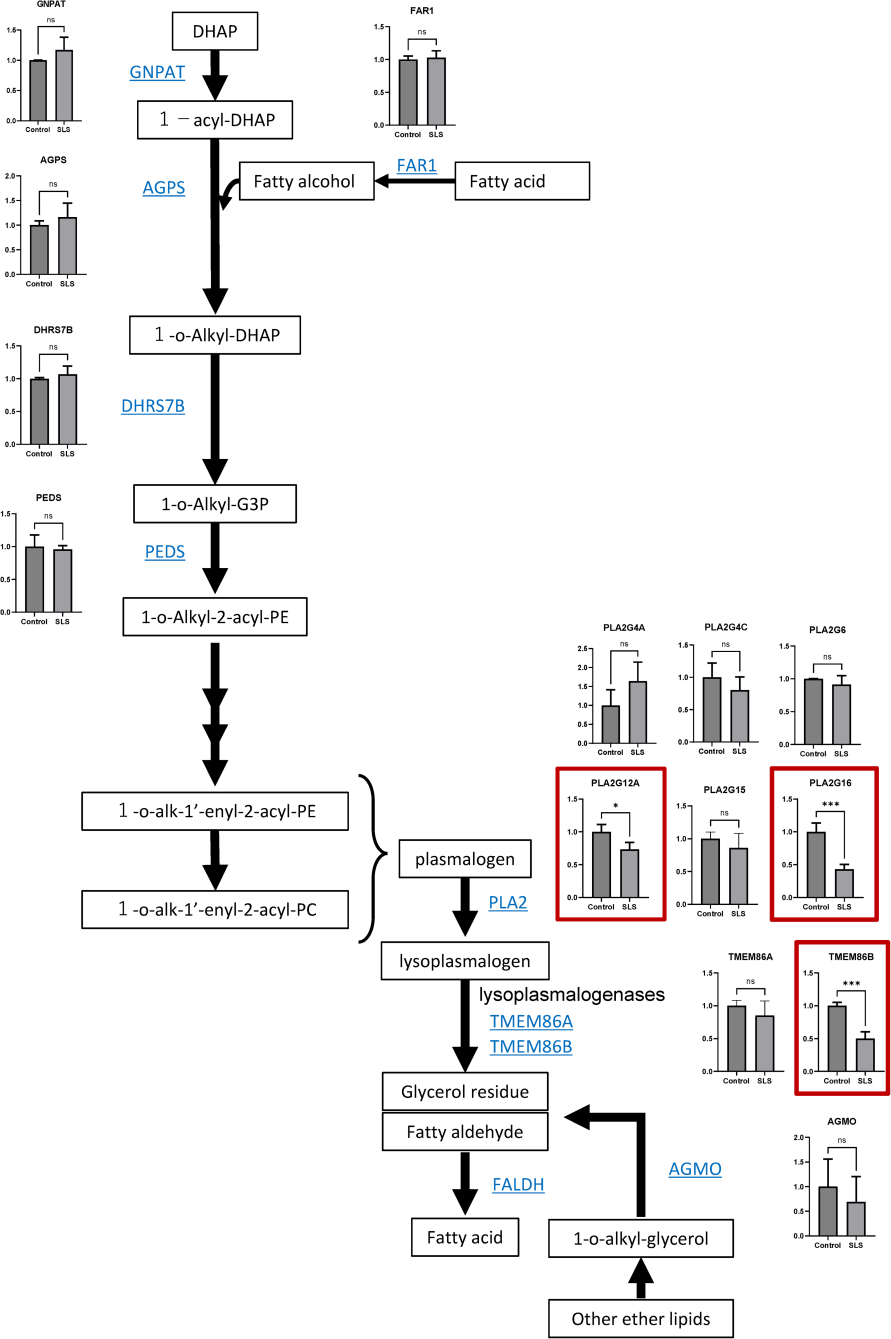
Decreased gene expression of plasmalogen hydrolytic enzymes and lysoplasmalogenases involved in FALDH substrate production. Fold changes of the mRNA expressions of each enzyme in oligospheres derived from the control and SLS‐iPSCs measured by RNA‐seq. *Y*‐axis: Fold change of the gene expressions as compared with the control (control = 1). Control: 201B7 and WD39; SLS: 1SLS12, 1SLS35, 2SLS1, 2SLS2 and 2SLS3. ns: Not significant, *p < 0.05, **p < 0.01, ***p < 0.001; unpaired *t*‐test. Significant differences in gene expression between the control and SLS are marked with red squares.

As shown in Figure 4, expressions of enzymes involved in ether lipid metabolism were compared between oligospheres derived from SLS‐iPSCs and those derived from control iPSCs. Among the genes showing altered expression, the expressions of *PLA2G12A* and *PLA2G16*, the genes encoding plasmalogen hydrolytic enzymes, and of *TMEM86B*, the gene encoding lysoplasminogenase,^20^ were significantly reduced in the SLS as compared with control oligospheres. Expressions of other enzymes in the biosynthetic pathway of ether lipids, including plasmalogens, were also evaluated, including fatty acid reductase (FAR1), plasmanylethanolamine desaturase (PEDS), glyceronephosphate *O*‐acyltransferase (GNPAT), alkylglycerone phosphate synthase (AGPS); alkyl/acyl DHAP reductase (DHRS7B), and alkylglycerol monooxygenase (AGMO). We found that the expression levels of these enzymes in the SLS‐oligospheres and control‐oligospheres were comparable.

## 4 DISCUSSION

In this report, we demonstrated, by LC–MS based phospholipid profiling focusing on phosphatidylcholine and phosphatidylethanolamine species, the accumulation of ether phospholipids in iPSCs, iPSC‐derived neurospheres, and oligospheres derived from SLS patients. The results are consistent with previously reported results showing accumulation of ether lipids in the postmortem brain tissue of a patient of SLS. Therefore, iPSCs, iPSC‐derived neurospheres, and oligospheres established from patients with SLS could prove to be useful tools for studying the pathophysiology in the central nervous system in patients with SLS.

We showed decreased expressions of plasmalogen‐hydrolyzing enzymes (i.e., PLA2G12A, and PLA2G16) and lysoplasmalogenase, which catalyzes plasmalogens to glycerophospho‐ethanolamine and fatty aldehyde,^20^ in the iPSCs, iPSC‐derived neurospheres, and oligospheres. Since lysoplasmalogenase is immediately upstream of FALDH, and PLA2G12A and PLA2G16 are immediately upstream of lysoplasmalogenase in the degradation pathway of ether phospholipids, decreased expressions of these enzymes could have contributed to an increased level of ether phospholipids in the cells established from the SLS patients. The detailed mechanism is unknown. However, it seems reasonable that when FALDH is not functioning, its upstream metabolites are increased, thereby causing negative feedback to these enzymes. Patient 1 shows more severe phenotypes than Patient 2. We consider the possibility that Patient 1 and Patient 2 differ in their ability to metabolize toxic substances that accumulate when FALDH is not functioning.

Accumulation of ether phospholipids was observed in iPSCs, neurospheres, and oligospheres—this dominance of phosphatidylcholine species over phosphatidyl‐ethanolamine species may be consistent with the previously reported results of the postmortem brain lipid analysis of an SLS patient.

Recently, the accumulation of plasmalogens, a major subclass of ether phospholipids, has been documented in the cells of patients with a pathogenic variant of the *FAR1* gene encoding the enzyme Fatty Acyl‐CoA Reductase 1, which converts acyl‐CoA to fatty alcohol. These patients with the gain‐of‐function variant p. Arg480Ser in FAR1 exhibit spastic paresis, reminiscent of SLS (MIM#619338). The neurological phenotype of *FAR1* gain‐of‐function (GOF) variants associated with elevated ether phospholipids supports the notion that ether phospholipids levels must be tightly controlled in the nervous system. Of interest, complete loss‐of‐function *FAR1* mutations decrease the levels of plasmalogen, a type of ether phospholipids and are associated with severely delayed psychomotor development, growth retardation with microcephaly, and seizures. This observation suggests that the health of the CNS, and probably the synthesis and maintenance of myelin, depends heavily on maintenance of the ether phospholipids at appropriate levels.

We expect that the in vitro model of SLS presented will be valuable in the research of the pathophysiology and development of SLS in the absence of a good mouse model, presumably due to interspecies differences in the neurological symptoms due to structural and functional divergence of the aldehyde dehydrogenase (ALDH) family between humans and mice.^6^

It is common for lipid components to differ greatly from cell type to cell type, even if they are derived from the same individual. However, when patient‐derived iPSCs, neurospheres, and oligospheres were compared with those derived from healthy individuals, the ratios of the phosphatidylcholine‐type ether lipids and phosphatidylethanolamine type ether lipids increased in all cell types derived from SLS patients. This result is consistent with previous reports suggesting that accumulation of ether phospholipids might occur in SLS patients.^6^, ^21^

Furthermore, these results suggest that the use of iPSCs, rather than neural and oligodendrocyte lineage cells, may be beneficial for further analysis of lipid accumulation observed in SLS patients. iPSCs are infinitely proliferating cells that can be easily used, and the possibility of using them for the analysis of drug scalability to examine drugs will be a major step forward in the development of a therapeutic agent for SLS.

## 5 CONCLUSIONS

In the present study, we established an in vitro cellular disease model by generating iPSCs from two SLS patients and further differentiating these cells into oligodendrocyte lineage cells. We documented an accumulation of ether phospholipids in these cells. This lipid profile partially recapitulated the finding of accumulation of ether phospholipids in the postmortem brain of an SLS patient reported previously. Accumulation of ether phospholipids was observed not only in oligodendrocyte lineage cells but also in neural cells and iPSCs.

## CONFLICT OF INTEREST STATEMENT

The authors declare no conflicts of interest.

## Supporting information


**Supplementary Figure S1.** Characterization of SLS patient‐derived iPSCs. Control and SLS‐derived iPSCs showed a typical colony morphology and both showed a normal chromosomal karyotype. Bar: 200 μm.

**Supplementary Table S1.** MRM transitions were used for the LC‐MRM‐MS phospholipid analysis. All MRMs were performed in positive ionization polarity.
