# Supplementary figures and images for: Accumulation of ether phospholipids in induced pluripotent stem cells and oligodendrocyte‐lineage cells established from patients with Sjögren‐Larsson syndrome
Source: Congenit Anom (Kyoto). 2024 Dec 1;65(1):e12587. doi: 10.1111/cga.12587 (PMC11608845; doi:10.1111/cga.12587)

Supplementary Figure 1. Characterization of SLS patient-derived iPSCs.

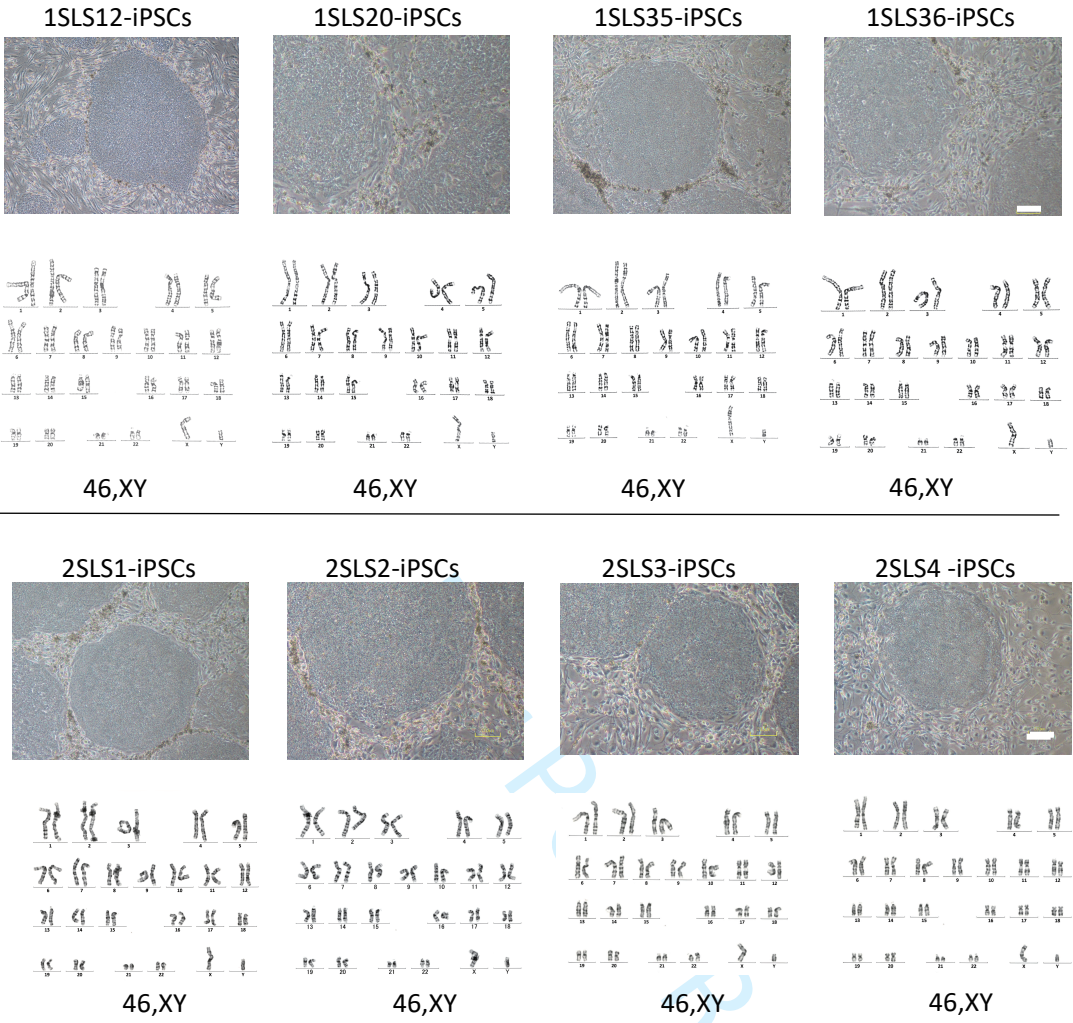

Supplement: Supplementary file 1 — Supplementary Figure S1. Characterization of SLS patient‐derived iPSCs. Control and SLS‐derived iPSCs showed a typical colony morphology and both showed a normal chromosomal karyotype. Bar: 200 μm. [file CGA-65-0-s001.pdf]
