# Supplementary material for: Accumulation of ether phospholipids in induced pluripotent stem cells and oligodendrocyte‐lineage cells established from patients with Sjögren‐Larsson syndrome
Source: Congenit Anom (Kyoto). 2024 Dec 1;65(1):e12587. doi: 10.1111/cga.12587 (PMC11608845; doi:10.1111/cga.12587)
Supplement: Supplementary file 2 — Supplementary Table S1. MRM transitions were used for the LC‐MRM‐MS phospholipid analysis. All MRMs were performed in positive ionization polarity. [file CGA-65-0-s002.docx]

Supplementary Table 1.

MRM transitions used for LC-MRM-MS phospholipid analysis. All MRM were performed in positive ionization polarity.

| Target | Q1 m/z | Q3 m/z |
| --- | --- | --- |
| LPC_14:0 | 468.3 | 184.1 |
| LPC_16:0 | 496.4 | 184.1 |
| LPC_16:1 | 494.3 | 184.1 |
| LPC_18:0 | 524.4 | 184.1 |
| LPC_18:1 | 522.4 | 184.1 |
| LPC_18:2 | 520.4 | 184.1 |
| LPC_20:1 | 550.4 | 184.1 |
| LPC_20:2 | 548.4 | 184.1 |
| LPC_20:3 | 546.4 | 184.1 |
| LPC_20:4 | 544.4 | 184.1 |
| LPC_22:3 | 574.4 | 184.1 |
| LPC_22:4 | 572.4 | 184.1 |
| LPC_22:5 | 570.4 | 184.1 |
| LPC_22:6 | 568.4 | 184.1 |
| LPE_16:0 | 454.3 | 313.3 |
| LPE_16:1 | 452.3 | 311.3 |
| LPE_18:0 | 482.3 | 341.3 |
| LPE_18:1 | 480.3 | 339.3 |
| LPE_18:2 | 478.3 | 337.3 |
| LPE_20:1 | 508.3 | 367.3 |
| LPE_20:2 | 506.3 | 365.3 |
| LPE_20:3 | 504.3 | 363.3 |
| LPE_20:4 | 502.3 | 361.3 |
| LPE_22:1 | 536.4 | 395.4 |
| LPE_22:2 | 534.4 | 393.3 |
| LPE_22:3 | 532.3 | 391.3 |
| LPE_22:4 | 530.3 | 389.3 |
| LPE_22:5 | 528.3 | 387.3 |
| LPE_22:6 | 526.3 | 385.3 |
| LPE_24:3 | 560.4 | 419.4 |
| LPE_24:4 | 558.4 | 417.3 |
| LPE_24:5 | 556.3 | 415.3 |
| LPE_24:6 | 554.3 | 413.3 |
| PC_28:0 | 678.6 | 184.1 |
| PC_28:1 | 676.5 | 184.1 |
| PC_30:0 | 706.6 | 184.1 |
| PC_30:1 | 704.6 | 184.1 |
| PC_30:2 | 702.6 | 184.1 |
| PC_32:0 | 734.6 | 184.1 |
| PC_32:1 | 732.6 | 184.1 |
| PC_32:2 | 730.6 | 184.1 |
| PC_32:3 | 728.6 | 184.1 |
| PC_32:4 | 726.5 | 184.1 |
| PC_34:0 | 762.6 | 184.1 |
| PC_34:1 | 760.6 | 184.1 |
| PC_34:2 | 758.6 | 184.1 |
| PC_34:3 | 756.6 | 184.1 |
| PC_34:4 | 754.6 | 184.1 |
| PC_34:5 | 752.5 | 184.1 |
| PC_36:0 | 790.6 | 184.1 |
| PC_36:1 | 788.6 | 184.1 |
| PC_36:2 | 786.6 | 184.1 |
| PC_36:3 | 784.6 | 184.1 |
| PC_36:4 | 782.6 | 184.1 |
| PC_36:5 | 780.6 | 184.1 |
| PC_36:6 | 778.6 | 184.1 |
| PC_36:7 | 776.5 | 184.1 |
| PC_38:1 | 816.6 | 184.1 |
| PC_38:2 | 814.6 | 184.1 |
| PC_38:3 | 812.6 | 184.1 |
| PC_38:4 | 810.6 | 184.1 |
| PC_38:5 | 808.6 | 184.1 |
| PC_38:6 | 806.6 | 184.1 |
| PC_38:7 | 804.6 | 184.1 |
| PC_40:1 | 844.7 | 184.1 |
| PC_40:2 | 842.6 | 184.1 |
| PC_40:3 | 840.6 | 184.1 |
| PC_40:4 | 838.6 | 184.1 |
| PC_40:5 | 836.6 | 184.1 |
| PC_40:6 | 834.6 | 184.1 |
| PC_40:7 | 832.6 | 184.1 |
| PC_40:8 | 830.6 | 184.1 |
| PC_O-28:0 | 664.5 | 184.1 |
| PC_O-28:1 | 662.5 | 184.1 |
| PC_O-30:0 | 692.6 | 184.1 |
| PC_O-30:1 | 690.6 | 184.1 |
| PC_O-30:2 | 688.6 | 184.1 |
| PC_O-32:0 | 720.6 | 184.1 |
| PC_O-32:1 | 718.6 | 184.1 |
| PC_O-32:2 | 716.6 | 184.1 |
| PC_O-34:0 | 748.6 | 184.1 |
| PC_O-34:1 | 746.6 | 184.1 |
| PC_O-34:2 | 744.6 | 184.1 |
| PC_O-34:3 | 742.6 | 184.1 |
| PC_O-34:4 | 740.6 | 184.1 |
| PC_O-36:0 | 776.5 | 184.1 |
| PC_O-36:1 | 774.6 | 184.1 |
| PC_O-36:2 | 772.6 | 184.1 |
| PC_O-36:3 | 770.6 | 184.1 |
| PC_O-36:4 | 768.6 | 184.1 |
| PC_O-36:5 | 766.6 | 184.1 |
| PC_O-36:6 | 764.6 | 184.1 |
| PC_O-38:0 | 804.6 | 184.1 |
| PC_O-38:2 | 800.6 | 184.1 |
| PC_O-38:3 | 798.6 | 184.1 |
| PC_O-38:4 | 796.6 | 184.1 |
| PC_O-38:5 | 794.6 | 184.1 |
| PC_O-38:6 | 792.6 | 184.1 |
| PC_O-38:7 | 790.6 | 184.1 |
| PC_O-40:2 | 828.6 | 184.1 |
| PC_O-40:3 | 826.6 | 184.1 |
| PC_O-40:4 | 824.6 | 184.1 |
| PC_O-40:5 | 822.6 | 184.1 |
| PC_O-40:6 | 820.6 | 184.1 |
| PC_O-40:7 | 818.6 | 184.1 |
| PE_30:0 | 664.6 | 523.6 |
| PE_30:1 | 662.6 | 521.6 |
| PE_32:0 | 692.6 | 551.6 |
| PE_32:1 | 690.6 | 549.6 |
| PE_32:2 | 688.6 | 547.6 |
| PE_32:3 | 686.5 | 545.5 |
| PE_34:0 | 720.6 | 579.6 |
| PE_34:1 | 718.6 | 577.6 |
| PE_34:2 | 716.6 | 575.6 |
| PE_34:3 | 714.6 | 573.6 |
| PE_34:4 | 712.5 | 571.5 |
| PE_36:1 | 746.6 | 605.6 |
| PE_36:2 | 744.6 | 603.6 |
| PE_36:3 | 742.6 | 601.6 |
| PE_36:4 | 740.6 | 599.6 |
| PE_36:5 | 738.6 | 597.6 |
| PE_38:1 | 774.6 | 633.6 |
| PE_38:2 | 772.6 | 631.6 |
| PE_38:3 | 770.6 | 629.6 |
| PE_38:4 | 768.6 | 627.6 |
| PE_38:5 | 766.6 | 625.6 |
| PE_38:6 | 764.6 | 623.6 |
| PE_38:7 | 762.6 | 621.6 |
| PE_38:8 | 760.6 | 619.6 |
| PE_40:2 | 800.6 | 659.6 |
| PE_40:3 | 798.6 | 657.6 |
| PE_40:4 | 796.6 | 655.6 |
| PE_40:5 | 794.6 | 653.6 |
| PE_40:6 | 792.6 | 651.6 |
| PE_40:7 | 790.6 | 649.6 |
| PE_40:8 | 788.6 | 647.6 |
| PE_O-32:0 | 678.6 | 537.6 |
| PE_O-32:1 | 676.6 | 535.6 |
| PE_O-34:0 | 706.6 | 565.6 |
| PE_O-34:1 | 704.6 | 563.6 |
| PE_O-34:2 | 702.6 | 561.6 |
| PE_O-36:1 | 732.6 | 591.6 |
| PE_O-36:2 | 730.6 | 589.6 |
| PE_O-36:3 | 728.6 | 587.6 |
| PE_O-36:4 | 726.6 | 585.6 |
| PE_O-36:5 | 724.6 | 583.6 |
| PE_O-38:1 | 760.6 | 619.6 |
| PE_O-38:2 | 758.6 | 617.6 |
| PE_O-38:3 | 756.6 | 615.6 |
| PE_O-38:4 | 754.6 | 613.6 |
| PE_O-38:5 | 752.6 | 611.6 |
| PE_O-38:6 | 750.6 | 609.6 |
| PE_O-38:7 | 748.6 | 607.6 |
| PE_O-40:3 | 784.6 | 643.6 |
| PE_O-40:4 | 782.6 | 641.6 |
| PE_O-40:5 | 780.6 | 639.6 |
| PE_O-40:6 | 778.6 | 637.6 |
| PE_O-40:7 | 776.6 | 635.6 |
